# Deep learning-based ovarian cancer subtypes identification using multi-omics data

**DOI:** 10.1186/s13040-020-00222-x

**Published:** 2020-08-24

**Authors:** Long-Yi Guo, Ai-Hua Wu, Yong-xia Wang, Li-ping Zhang, Hua Chai, Xue-Fang Liang

**Affiliations:** 1 Second School of Clinical Medicine, Guangzhou University of Chinese Medicine, Guangzhou, 510020 China; 2 Center for Reproductive Medicine, Guangdong Hospital of Traditional Chinese Medicine, Guangzhou, 510120 China; 3 School of Data and Computer Science, Sun Yat-sen University, Guangzhou, 510000 China

**Keywords:** Ovarian cancer, Deep learning, Multi-omics

## Abstract

**Background:**

Identifying molecular subtypes of ovarian cancer is important. Compared to identify subtypes using single omics data, the multi-omics data analysis can utilize more information. Autoencoder has been widely used to construct lower dimensional representation for multi-omics feature integration. However, learning in the deep architectures in Autoencoder is difficult for achieving satisfied generalization performance. To solve this problem, we proposed a novel deep learning-based framework to robustly identify ovarian cancer subtypes by using denoising Autoencoder.

**Results:**

In proposed method, the composite features of multi-omics data in the Cancer Genome Atlas were produced by denoising Autoencoder, and then the generated low-dimensional features were input into *k*-means for clustering. At last based on the clustering results, we built the light-weighted classification model with L1-penalized logistic regression method. Furthermore, we applied the differential expression analysis and WGCNA analysis to select target genes related to molecular subtypes. We identified 34 biomarkers and 19 KEGG pathways associated with ovarian cancer.

**Conclusions:**

The independent test results in three GEO datasets proved the robustness of our model. The literature reviewing show 19 (56%) biomarkers and 8(42.1%) KEGG pathways identified based on the classification subtypes have been proved to be associated with ovarian cancer. The outcomes indicate that our proposed method is feasible and can provide reliable results.

## Background

Ovarian cancer is one of the most common gynecologic cancers in the world that rank third after cervical and uterine cancer, and its mortality rate is high. Therefore, it is very important to know more about the ovarian cancer heterogeneity for choosing different treatment responses and predicting patients’ clinical outcomes. One way to research the heterogeneity is identifying different molecular subtypes in ovarian cancer, and many machine learning methods have been proposed for solving this problem [1, 2]. With the development of biological sequencing technology, different kinds of genomic data were utilized for ovarian cancer research: Liu et al. used hierarchical clustering to identify poor prognostic ovarian cancer with mRNA data [3]. Penyige et al. used miRNA expression data for differential expression analysis to find ovarian cancer-associated biomarkers [4]. Bodelon et al. identify ovarian cancer subtypes using DNA methylation profiling with nonnegative matrix factorization (NMF) clustering algorithm [5], Macintyre et al. proved the copy number is related to the ovarian cancer survival and the probability of platinum-resistant relapse by using NMF mixture modeling [6]. Although these data provide different sight on ovarian cancer research, the results is easy to be affected by the noise and missing data in one type of omics data, and the single omics-data can only provide limited information for ovarian cancer research.

In recently years, the Cancer Genome Atlas (TCGA) shared multiple omics data from tens of thousands of samples from 38 cancer types, making it possible to use multi-omics data for cancer subtype identification. Considering the high dimensional features in multi-omics data integration, the traditional methods such as *k*-means cannot achieve satisfied performance for clustering. Therefore, many different unsupervised learning methods were designed to deal with high dimensional features. Witten and Tibshirani proposed a Sparse K-means (SparseK) for cancer subtype identification by using an adaptively chosen subset of the features [7]; Xie et al. used iCluster to identify novel molecular subtypes of high-grade serous ovarian cancer by the integration of gene expression and proteomics data [8]. Another way to improve the clustering performance is to reduce the dimensionality of features before using clustering method. Principal component analysis (PCA) is one of the most widely used method for dimensionality reduction. In Aelex’s study, PCA was applied to reconstruct the gene features and the clustering method *k*-means was used for distinguishing subtypes of breast cancer using the reconstructed features [9]. Nevertheless, PCA is one of the linear dimensionality reduction method, which means the function mapping from high-dimensional space to low-dimensional space is linear. However, in many cases, linear mapping may not get the desired results. The kernel PCA (KPCA) was proposed for solving this problem that can be seen as an extension of PCA using additional kernel function. Compared to PCA, the performance of processing nonlinear data in KPCA was improved. Ha et al. used the logistic regression method with the features reconstructed by KPCA for cancer classification [10]. With the development of deep learning technology, Autoencoder (AE) was designed to construct lower dimensional representation for integrating the multi-omics data. Chaudhary et al. employed AE to reconstruct low dimensional representation from three types of omics data (mRNA, miRNA and DNA methylation), and input them into *k*-means to identify different molecular subtypes of the liver hepatocellular carcinoma [11]. The result in [11] demonstrates the advanced performance of deep learning on high-dimensional feature clustering. Nevertheless, the generated lower dimensional representation was easy to be affected by the noise in the input data because the input and output are equal in AE framework. Due to the lack of robustness of AE, it is difficult to extract the most informative features from high-dimensional multi-omics data in practical applications.

Trying to solve this problem, we proposed a novel deep learning framework for integrating multi-omics data with denoising autoencoder (DAE), and then the generated features were input into *k*-means for clustering (DAE-kmeans). Compared to AE, DAE proposed by Vincent [12] can make the features learned by the model more robust by superimposing noise on the input. As we know, there are many variants of autoencoder, including denoising autoencoder, sparse autoencoder, convolutional Autoencoder and variational autoencoder (VAE). For sparse autoencoder, there are more nodes in the hidden layer than in the input layer, which increases the difficulty of calculation and are not used for dimension reduction. The convolutional autoencoder are more used to process images. Comparing with the DAE and VAE, they both have encoder and decoder blocks, but their purpose is different. DAE trained the input features in which some noise is added, for ensuring the network will not learn an identity mapping which are pointless. VAE belongs to one of the explicit distributed modeling technologies. If we want to model the input features into some distribution and want to know the parameters of the distribution, then VAE is a better choice. Hence DAE is used for to learn a more robust latent representation for features, and VAE is used where if want to learn the probability distribution of the input.

By using DAE, our proposed deep learning framework can obtain the most informative features which represent the multi-omics data, and then utilized the reconstructed features to identify the molecular subtypes by *k*-means. The results proved that compared with AE, our method achieved 6.2% higher silhouette score in clustering and could separate the ovarian cancer patients into different subtypes with more significant differences (*p*-value< 0.05). For reducing the number of features used in ovarian cancer subtype identification, we further build a light-weighted logistic regression classification model with mRNA features. And the results in three independent datasets (GSE26712, GSE53963 and GSE63885) proved the robustness of our classification model (all *p*-values between the classified subtypes< 0.05).

## Methods

### Datasets

In this study we utilized the multi-omics ovarian data for training and three datasets in GEO were used as the independent tests. The details about these four datasets were introduced in following:

#### TCGA dataset

- We downloaded the multi-omics ovarian cancer data from TCGA public datasets((https://portal.gdc.cancer.gov). The *R* package *TCGA-assemble2* [13] was used for data collection and we obtained 298 samples concluded three types of omics data: mRNA-seq data (UNC Illumina HiSeq_RNASeq V2), miRNA-seq data (BCGSC Illumina HiSeq) and copy number variation (CNV) data (BROAD-MIT Genome wide SNP_6). All these data were obtained from the TCGA level 3 data. And the CNV feature was extracted by averaging the copy numbers of all CNV variations on one gene. After that the features and samples which missing more than 20% would be excluded. For the remaining data, the missing values were imputed based on the median values by using *R* package “*imputeMissings*” [14].

#### Test datasets

- In GSE26712 we downloaded the RNA-seq and the clinical information of 185 ovarian cancer patients shared from Surgical Oncology Research Lab of Boston, and in GSE32062 we got 260 ovarian cancer case samples offered by Obstetrics and Gynecology, Niigata University. GSE53963 contains mRNA information of 174 samples from UCLA School of Medicine. All of these test datasets can be downloaded in Gene Expression Omnibus (GEO, https://www.ncbi.nlm.nih.gov)

### The architecture of proposed deep learning framework

**Fig. 1:**
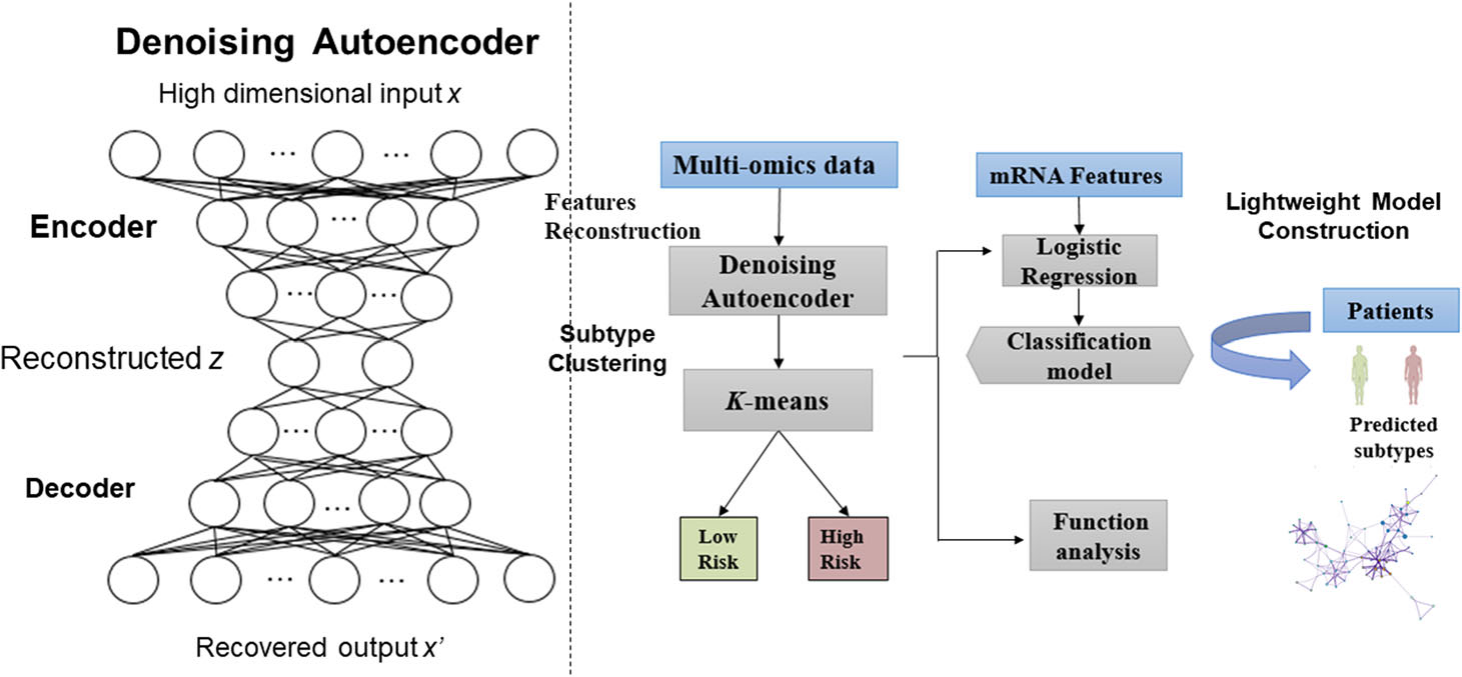
The architecture of proposed deep learning framework for identifying ovarian cancer subtypes

In Fig. 1 we show the architecture of proposed deep learning framework, firstly the multi-omics ovarian cancer features *x* (mRNA, miRNA and CNV) are inputted into the DAE for generating the low dimensional representation *z*. And then the reconstructed features *z* are used to cluster the patients using the *k*-means. Based on the clustered subtypes from *k*-means, we further built the light-weighted logistic regression classification model with mRNA expression data to reducing the features required for patients’ classification. The available code of this deep learning framework was shared in https://github.com/Hua0113/DAE_km.

### Denoising autoencoder for dimensionality reduction

The Autoencoder (AE) is one of the deep neural network that used to copy its input to its output, and supposing the bottleneck layer *z* can be seen as the represent of the input features. AE consists of two parts: the encoder part *z* = *f*_*e*_(*x*) and the decoder part *x*^′^ = *f*_*d*_(*z*), and the loss function of AE can be expressed as:

1 $$ {l}_{AE}={\left\Vert x-{x}^{\prime}\right\Vert}_2^2={\left\Vert x-{f}_d\left({f}_e(x)\right)\right\Vert}_2^2 $$

Different as traditional AE, DAE constructs partially damaged data by adding noise to the input data, and restores it to the original input data by encoding and decoding, which make the deep neural network has the ability to identify useful features, the new generated input $\overset{\sim }{x}$ can be expressed as:

2 $$ \overset{\sim }{x}={q}_{\mathcal{D}}\left(\overset{\sim }{x}|x\right) $$

Where ${q}_{\mathcal{D}}$ represents the stochastic mapping. And then the corrupted input $\overset{\sim }{x}$ is inputted to a deep neural network for encoding and decoding with the same process of the standard autoencoder. The loss of the DAE is written as:

3 $$ {l}_{AE}={\left\Vert x-{x}^{\prime}\right\Vert}_2^2={\left\Vert x-{f}_d\left({f}_e\left(\overset{\sim }{x}\right)\right)\right\Vert}_2^2 $$

In our study, the DAE is a 7 layers deep neural network (input, output, and 5 hidden layers), the nodes of the 5 hidden layers were set 200, 50, 2, 50 and 200 respectively. In each layer, we used tanh as the nonlinear activation function and the DAE was trained by back-propagation via the Adam optimizer. The learning rate of our model was set 0.001, the batch size was set 256 and the epoch was set 100. These parameters were selected for maximizing the silhouette score in OV.

### K-means clustering using reconstructed features

The DAE was used to construct the low dimensional features of the multi-omics data from the bottleneck layer. After obtaining the reconstructed features, K-means method was used for ovarian cancer subtypes clustering. We determined the optimal number of clusters with silhouette score [15]. We test the *k* from [2, 8] and set *k* = 2 because of the highest silhouette score.

### Logistic regression method for subtypes classification

After obtaining the labels clustered by *k*-means, we built a light-weighted mRNA model for reducing the number of genes needed to identify cancer subtypes by using logistic regression algorithm. Here we used the mRNA omics data as the features *X* and the subtypes clustered based on our DAE-kmeans framework as the label *Y*. Defining ${x}_i^{mrna}\in X$ represent the mRNA features of patient i, *y*_*i*_ ∈ *Y* is the subtypes of the patient i (Low = 0, High =1), *β* is the coefficient vector, the logistic regression can be expressed as

4 $$ p\left({y}_i=1\right)=\frac{\mathit{\exp}\left({\beta}_0+\beta {x}_i^{mrna}\right)}{1+\mathit{\exp}\left({\beta}_0+\beta {x}_i^{mrna}\right)} $$

Where *p*(*y*_*i*_ = 1) represent the probability that the patient *i* belongs to the high-risk group. The log-likelihood function of logistic model is written as:

5 $$ l\left(\beta \right)=\sum \limits_{i=1}^n\left\{{y}_i\ln \left({p}_i\right)+\left(1-{y}_i\right)\ln \left(1-{p}_i\right)\right\} $$

Many different regularizations are used to improve the generalization ability of the model [16, 17]. Considering to reduce the number of features in constructing the classification model, the L1 regularization is combined with logistic regression method:

6 $$ \beta =\mathrm{agr}\ \max \left[\sum \limits_{i=1}^n\left\{{y}_i\mathit{\ln}\left({p}_i\right)+\left(1-{y}_i\right)\mathit{\ln}\left(1-{p}_i\right)\right\}-\lambda g\left(\beta \right)\right] $$

Here all the samples in TCGA were used as training data, and the classification model obtained with logistical regression method was evaluated in three ovarian cancer mRNA datasets from GEO as the independent test.

### Evaluations of ovarian cancer subtypes identification

We implemented different methods for comparing the performances of different cluster methods: k-means*,* hierarchical clustering, k-means using the reconstructed features by PCA (PCA-kmeans), SparseK, iCluster, k-means using the reconstructed features by KPCA (KPCA- kmeans), k-means using the reconstructed features by AE (AE-kmeans) and DAE-kmeans. The silhouette score is used to measure the cluster performance and the log rank *p*-value to measure the differences of the different subtypes of cancers. The higher silhouette score means the method achieved better performance for clustering, and the lower log-rank p-value means the greater differences in cancer subtypes.

### Functional analysis

Based on the identified subtypes of ovarian cancer, differentially expressed gene (DEG) analysis was applied by using *R* package *DESeq2* [18], which the genes with corrected *p*-values < 0.05 and |log2 fold change| ≥ 1 were seen as the DEGs. And we also applied WGCNA analysis by the *R* package *WGCNA* [19], for identifying function modules and genes related to ovarian cancer subtypes. In WGCNA analysis, we selected the unsigned network, the least genes in each module was set 30, and height cut-off parameter used to merge similar modules was set 0.25. The genes in each module which have a higher relevance score (> 0.5) were defined as the hub genes (HGs). At last the genes which both belong to DEGs and HGs are seen as candidate genes which highly related to ovarian cancer. And the enriched pathways were obtained by these genes based on the KOBAS online tool [20].

## Results

In Table 1 we show the clustering performances obtained from different methods by using ovarian cancer multi-omics data which contained mRNA, miRNA and CNV. We used the silhouette scores and Davies Bouldin scores (DBI) to evaluate the clustering performances of the methods. It is obviously that without any dimensionality reduction method, *K*-means achieved lowest silhouette score and highest DBI among these methods. And the methods based on traditional dimensionality reduction methods (PCA, KPCA) performed only better than *k*-means and hierarchical clustering, but worse than SparseK, iCluster and two deep learning-based methods. The results in Table 1 prove the power of deep learning, and DAE-kmeans perform best than any other methods indicated the superiority of our method.

**Table 1:** The clustering performances obtained by different methods in ovarian cancer

	silhouette scores	DBI
*K*-means	0.165	1.859
Hierarchical clustering	0.310	1.594
PCA- kmeans	0.378	1.502
KPCA-kmeans	0.475	0.702
SparseK	0.513	0.681
iCluster	0.528	0.657
AE-kmeans	0.549	0.621
DAE-kmeans	0.583	0.562

In Table 2 we give the clustering performance comparison using different type of omics data. The results indicated that when using single type of omics data, the mRNA performed best with the silhouette score 0.550, and the CNV achieved worst performance with silhouette score value of 0.509. The miRNA performed better than CNV but worse than mRNA. It is obviously that clustering using multi-omics in our deep learning framework achieved 6% higher silhouette score and 7.41% lower DBI, compared with which obtained by using mRNA data.

**Table 2:** The clustering performance comparison using different type of omics data

	Features	silhouette score	DBI
mRNA	20,502	0.550	0.607
miRNA	1870	0.536	0.644
CNV	23,606	0.509	0.713
Multi-omics	45,978	0.583	0.562

**Fig. 2:**
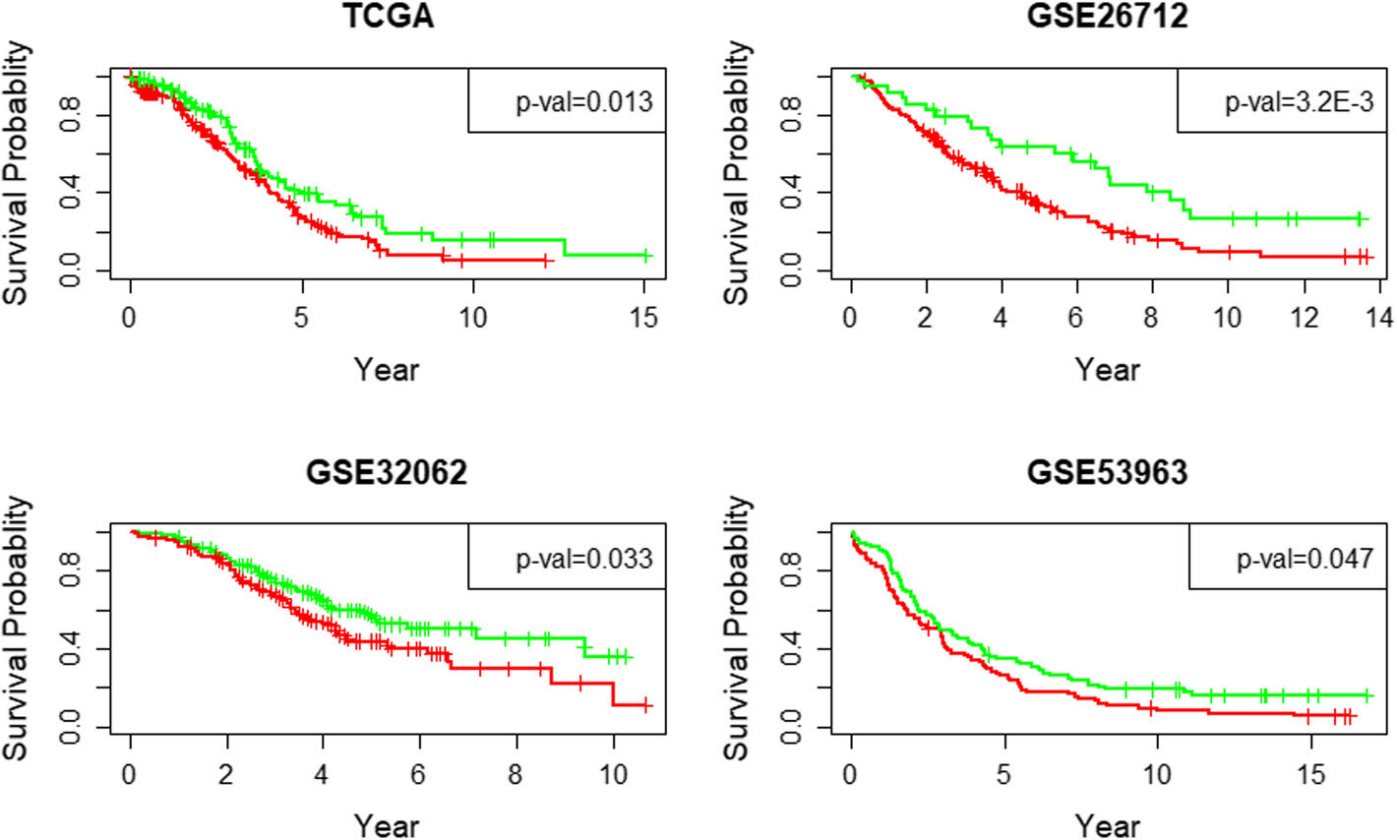
The survival curves drawn based on the identified subtypes in four ovarian cancer datasets. The green lines represent the patients have the high survival probability and the red lines mean the survival probability of the patients in this group is lower

Based on the labels clustered by DAE-kmeans, we built an L_1_-normalized logistic regression model to identify cancer subtypes with less features. Based on the final classification model, 134 mRNA features were selected and three GEO datasets were used as the independent tests to prove the robustness of the built classifier. The KM survival curves drawn based on the clustered result in OV dataset and the predicted results in three GEO datasets are given in Fig. 2. The result in Table 3 show that all the *p*-values are less than 0.05, which indicated that the differences between the different subgroups in every dataset are very significant.

**Table 3:** The performance of subtypes identification for the four ovarian cancer datasets

	Censored	Uncensored	Low Risk	High Risk	P-values
OV	122	176	111	187	0.013
GSE26712	56	129	82	103	3.2E-3
GSE32062	139	121	152	108	0.047
GSE53963	21	153	94	80	0.033

**Fig. 3:**
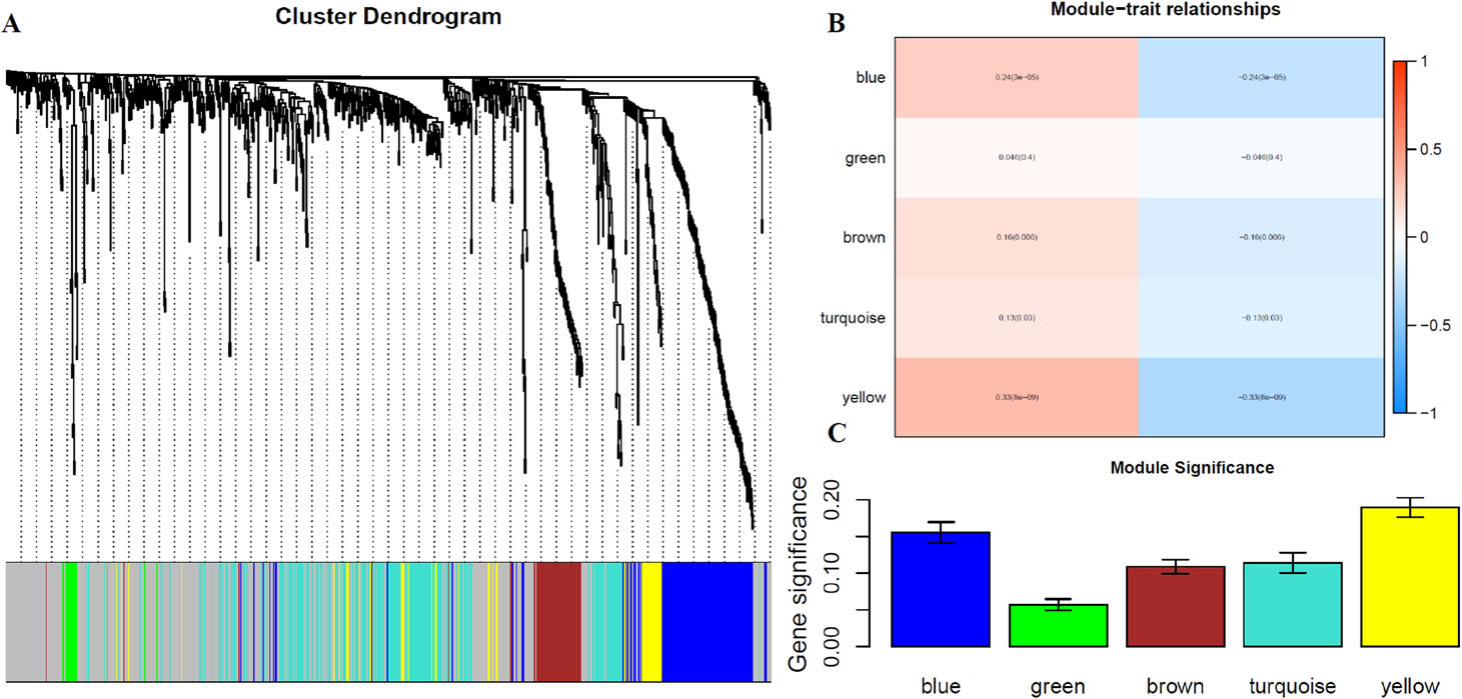
The results obtained by WGCNA in ovarian cancer dataset from TCGA: **a**. The gene dendrogram and identified modules in OV data; **b**. The correlation between the clustered modules and molecular subtypes; **c**. The average GS in each module in OV data

After obtaining the identified ovarian cancer subtypes in TCGA, we used the *R* package “*DESeq2*” to select the DEGs which p-value < 0.05 and |log2 fold change| ≥ 1, and 177 genes were selected as the candidate genes associated with ovarian cancer subtypes. And the *R* package “*WGCNA*” was applied to select the hub genes in the different function modules in ovarian cancer. The produced results were shown in Fig. 3. In WGCNA the genes with similar expression patterns were put into the same modules by average linkage clustering, and 5 different modules were clustered based on the histological grade of ovarian cancer dataset Fig. 3(a). The different clustered modules from WGCNA are represented by different colors. According to the features in each module, we computed the correlation between these modules and each phenotype Fig. 3(b), and the correlation between the genes and subtypes in these modules were used to measure the degree of correlation between the genes and ovarian subtypes (GS). The larger value represents the more significant affect to the function modules. The average GS in each module was shown in Fig. 3(c). At last 185 genes which GS > 0.5 were selected as the hub genes in ovarian cancer.

At last the genes which belong to both DEGs and hub genes are seen as the target genes which highly related to ovarian cancer, and finally 34 genes were selected (***ADH1B***, *BARX1*, *C7*, *CADPS*, ***CCL21***, *CFAP100*, *CFAP65*, ***COL11A1***, ***COL1A1***, ***COL2A1***, ***COL5A1***, *COL8A1*, ***COMP***, ***CXCL14***, *ECEL1*, *EFCAB1*, *FMO2*, ***FNDC1***, ***INHBA***, ***LRRC15***, *NKAIN4*, *OMD*, ***PLCB1***, *PLCXD3*, ***SERPINE1***, ***SFRP2***, ***SFRP4***, ***SNTN***, *SORCS*2, *SVEP1*, ***THBS2***, ***THBS4***, ***TUBA4B***, *VCAN*). Among these 34 genes, the literature reviewing shows that 19 genes (in bold) have been proved to be associated with the ovarian cancer. For example, the protein encoded by *ADH1B* is a member of the alcohol dehydrogenase family, and it has been proved to promote the mesothelial clearance and ovarian cancer infiltration [21]; *COL11A1* encodes one of the alpha chains of type XI collagen, and it promotes tumor progression and relates to ovarian cancer survival [22]; The overexpression of *FNDC1* was associated with cancer poor prognosis, and was identified as a potential biomarker in ovarian cancer treatment [23].

**Fig. 4:**
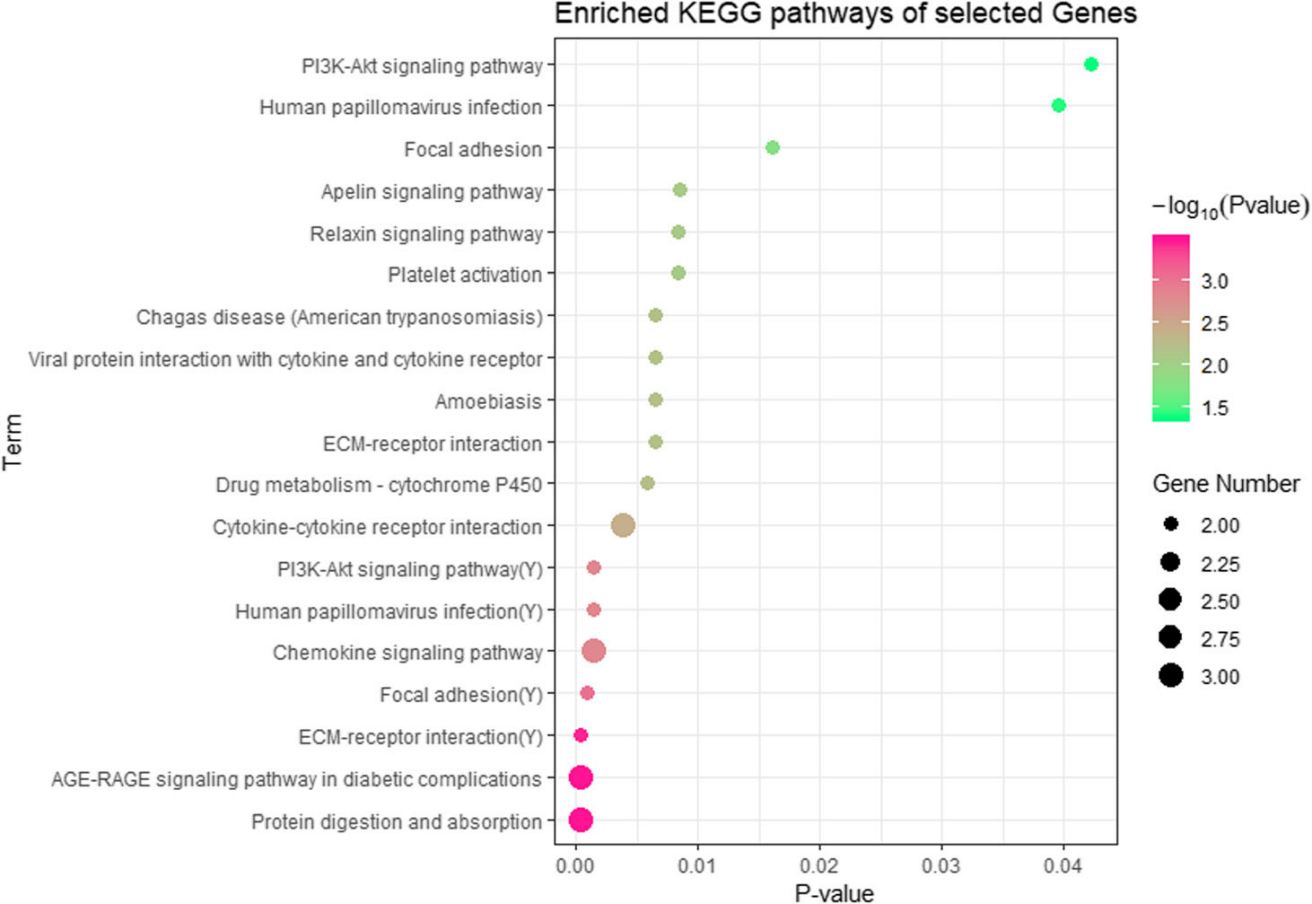
KEGG pathway enrichment analysis for 34 identified genes, the x-axis shows the *p*-value of each term and the y-axis shows the KEGG pathway terms. *(Y) means it is the pathways enriched in the yellow function module

After gene selection, we check distribution of these 34 genes in different modules, and the genes in different modules were enriched for KEGG (Kyoto Encyclopedia of Genes and Genomes) pathway analysis by using the online tool KOBAS (Table 4). 16 pathways which the corrected *p*-values < 0.05 and gene numbers > = 2 were identified to be related to the ovarian cancer subtypes (Fig. 4). Among these pathways, *ECM-receptor interaction*, *Human papillomavirus infection* and *PI3K-Akt signaling pathway* were both enriched in the blue and yellow function modules. We identified many ovarian cancer-related pathways including *PI3K-Akt* signaling pathway, *human papillomavirus infection* pathway. *PI3K-Akt signaling* pathway regulates the proliferation and survival of tumor cells, and its abnormal activity can not only lead to malignant transformation of cells, but also related to the migration of tumor cells [24]. In addition, the identified pathway about the human papillomavirus infection have been proved to be highly associated with ovarian cancer [25]. Moreover, we find some other cancer-related pathways including *ECM-receptor interaction*, *cytokine-cytokine receptor interaction*, and *Drug metabolism - cytochrome P450*.

**Table 4:** The distribution of selected genes and pathways in different modules

Module	Candidate Gene number	Enriched KEGG pathways
Blue	22	15
Green	0	0
Brown	5	0
Turquoise	1	0
Yellow	6	4

## Discussion

Though many studies for subtype identification of the OV patients by using different methods have been proposed, most reported OV subtype models have either no or very few independent tests as external validation. In this study we designed a novel deep learning-based framework for ovarian cancer subtype identification, and a logistic regression method was used to build the light-weighted classification model. Two ovarian cancer subtypes were founded by using multi-omics data in TCGA, and the result proved that these 2 different subtypes specific model proposed by our method is of direct clinical importance, and may be used for improving ovarian patients’ survival. Our research also extended the underlying prognosis related biological biomarkers based on these two risk groups. The results have proved the robustness and reliability of our model.

However, some caveats about our method are still worth discussion below: Firstly, TCGA samples have been reported are impure in a previous study [26]. The purity issue, along with the heterogeneous nature of ovarian cancer due to various risk factor, may influence the accuracy of our method. To further analysis the effects of risk factors on the ovarian cancer, in future work we will try to identify ovarian cancer subtypes with more clinical factors including the age and race of the cancer patients. Secondly, sample size is one of the biggest challenges in limiting bioinformatics methods for cancer subtype clustering, which calls for better strategies. Trying to solve this problem, transfer learning mechanism is considered in our framework. Thirdly, as we know, cancer images can reflect information about the impact of molecular changes on cancer cells and the aggressiveness of the disease, in next step we will integrate the multi-omics expression data and the information from the cancer image, and improve the model over time.

## Conclusions

It is important to know more about the ovarian cancer heterogeneity between different patients for choosing different treatment programs and predicting clinical outcomes. In this study we proposed a novel deep learning framework for integrating multi-omics data with denoising autoencoder for identifying the ovarian cancer subtypes. Two subtypes from the molecular level were identified in ovarian cancer, and the results show our proposed method is competitive and reliable. The method comparison results indicated our method out-performed than the traditional and deep learning-based methods. More importantly, the classification model was proved by three independent test datasets collected from GEO. All the *p*-values less than 0.05 show that the differences between the classified cancer subgroups are significant.

By combining the results in DEG and WGCNA analysis, we selected 34 target genes related to ovarian cancer. And using these 34 identified genes, 19 KEGG pathways were enriched including PI3K-Akt signaling pathway and human papillomavirus infection pathway. The literature reviewing show 19 (56%) biomarkers and 8(42.1%) KEGG pathways identified based on the classification subtypes have been proved to be associated with ovarian cancer. These results indicate that our proposed method is reliable and advanced.

## Data Availability

All the data analyzed during the current study are available in the TCGA and GEO datasets.

## Abbreviations

TCGA: The Cancer Genome Atlas
PCA: Principal component analysis
KPCA: Kernel principal component analysis
AE: Autoencoder
DAE: Denoising autoencoder
CNV: Copy number variation
DEG: Differentially expressed gene
HG: Hub gene
DBI: Davies Bouldin scores
KEGG: Kyoto Encyclopedia of Genes and Genomes
